# Supplementary material for: Health Patterns among Migrant and Non-Migrant Middle- and Older-Aged Individuals in Europe—Analyses Based on Share 2004–2017
Source: Int J Environ Res Public Health. 2021 Nov 16;18(22):12047. doi: 10.3390/ijerph182212047 (PMC8622058; doi:10.3390/ijerph182212047)
Supplement: Supplementary file 1 [file ijerph-18-12047-s001.zip › ijerph-1421981-supplementary.pdf]

Supplement

Table S1 Results of logistic regression models, including interaction terms (odds ratios, 95% confidence intervals and p-values)

Parameter	Limited Global Activity Limitation Index (GALI)			Depression			Poorer Self-rated Health		
	Odds Ratio	95% CI	p	Odds Ratio	95% CI	p	Odds Ratio	95% CI	p
(Intercept)	0.93	(0.85, 1.02)	0.120	0.58	(0.53, 0.65)	< .001	4.94	(4.46, 5.47)	< .001
Wave	1.02	(1.01, 1.03)	0.004	1.03	(1.01, 1.04)	< .001	1.02	(1.00, 1.03)	0.035
Migrant	1.30	(1.07, 1.59)	0.009	1.42	(1.14, 1.77)	0.002	1.13	(0.90, 1.42)	0.281
Age (between subjects)	0.94	(0.86, 1.02)	0.144	1.03	(0.93, 1.14)	0.526	0.92	(0.83, 1.01)	0.079
Female gender	0.94	(0.87, 1.02)	0.138	0.98	(0.89, 1.07)	0.628	0.95	(0.87, 1.04)	0.277
Area of Living									
Metropolitan (Reference)	1.00	-	-	1.00	-	-	1.00	-	-
Metroplitan area	0.99	(0.91, 1.07)	0.775	0.93	(0.85, 1.02)	0.107	0.92	(0.84, 1.00)	0.058
Large City	1.00	(0.94, 1.08)	0.909	0.96	(0.88, 1.03)	0.261	0.95	(0.88, 1.03)	0.211
Small city	0.98	(0.91, 1.05)	0.508	0.87	(0.81, 0.94)	< .001	1.02	(0.94, 1.10)	0.646
Rural	1.15	(1.08, 1.23)	< .001	0.89	(0.83, 0.96)	0.003	1.07	(0.99, 1.15)	0.084
Education									
lower/upper secondary (Reference)	1.00	-	-	1.00	-	-	1.00	-	-
post-secondary	0.74	(0.70, 0.77)	< .001	0.57	(0.54, 0.60)	< .001	0.60	(0.57, 0.64)	< .001
tertiary	0.53	(0.50, 0.56)	< .001	0.39	(0.37, 0.42)	< .001	0.33	(0.31, 0.36)	< .001
Household Size	1.00	(0.99, 1.02)	0.657	0.98	(0.96, 1.00)	0.096	1.00	(0.98, 1.02)	0.775
Age (within subject)	1.02	(1.00, 1.04)	0.032	0.99	(0.97, 1.01)	0.498	1.02	(1.00, 1.05)	0.064
wave * Migrant	0.99	(0.95, 1.04)	0.798	0.95	(0.90, 1.00)	0.048	1.03	(0.98, 1.08)	0.193
wave * Age	1.01	(0.99, 1.03)	0.294	0.99	(0.97, 1.02)	0.576	1.01	(0.99, 1.04)	0.218

Migrant * Age	0.90	(0.69, 1.18)	0.460	1.01	(0.74, 1.38)	0.948	1.01	(0.74, 1.38)	0.951
wave * Female	1.02	(1.00, 1.03)	0.033	1.01	(0.99, 1.03)	0.532	1.02	(1.00, 1.04)	0.106
Migrant * Female	1.11	(0.86, 1.43)	0.434	0.84	(0.63, 1.13)	0.255	1.41	(1.04, 1.93)	0.027
Age * Female	1.05	(0.94, 1.18)	0.354	1.08	(0.95, 1.23)	0.255	1.04	(0.92, 1.18)	0.493
Wave * Migrant * Age	1.02	(0.96, 1.08)	0.555	0.98	(0.91, 1.07)	0.694	0.99	(0.92, 1.06)	0.747
Wave * Migrant * Female	0.97	(0.92, 1.02)	0.260	1.04	(0.98, 1.12)	0.203	0.92	(0.86, 0.99)	0.018
Wave * Age * Female	0.99	(0.97, 1.01)	0.325	1.00	(0.97, 1.03)	0.808	1.00	(0.97, 1.02)	0.717
Migrant * Age * Female	1.06	(0.74, 1.50)	0.755	0.77	(0.50, 1.18)	0.223	0.89	(0.59, 1.33)	0.570
Wave * Migrant * Age * Female	0.99	(0.92, 1.07)	0.800	1.04	(0.93, 1.15)	0.494	1.04	(0.95, 1.14)	0.433
Number of observations	230,278			179,426			230,184		

To study the country-specific trends in GALI, depression and self-perceived health over time, we employed meta-analyses with random-effect models. Within each country subsample, one logistic regression model for each wave was calculated and the proportions of GALI, depression and self-perceived health predicted by migrant vs. non-migrant were estimated. These estimated proportions were used effect sizes for the meta-analyses. The resulting coefficients indicate the change (increase/decrease) in the proportion of our health indicators over time (per year) for migrants and non-migrants within each country. Meta-analyses were conducted using the R-package "metafor" (Viechtbauer, W. (2010). Conducting meta-analyses in R with the metafor package. Journal of Statistical Software, 36(3), 1-48. https://doi.org/10.18637/jss.v036.i03).
